# Involvement of mitogen activated protein kinase kinase 6 in UV induced transcripts accumulation of genes in phytoalexin biosynthesis in rice

**DOI:** 10.1186/1939-8433-6-35

**Published:** 2013-12-02

**Authors:** Dhammaprakash Pandhari Wankhede, Kundan Kumar, Pallavi Singh, Alok Krishna Sinha

**Affiliations:** National Institute of Plant Genome Research, Aruna Asaf Ali Road, New Delhi, India; Department of Biological Sciences, Birla Institute of Technology and Science-Pilani, KK Birla Goa Campus, Zuarinagar, Goa India

**Keywords:** MAPKK, *Magnaporthe oryzae*, Phytoalexins, Rice, UV

## Abstract

**Background:**

Ultra violet radiation leads to accumulation of phytoalexins (PA) in rice (*Oryza sativa*) which are typically accumulated when the plants are infected with rice blast pathogen *Magnaporthe oryzae*. Although extensive works have been done in elucidating phytoalexin biosynthesis, UV stress signal transduction leading to accumulations of rice phytoalexin is largely unknown.

**Results:**

In the present study, the involvement of mitogen activated protein kinase (MAPK) cascade has been shown in UV induced regulation of genes in phytoalexin biosynthesis in rice. UV induced activation of MAPK and expression of PA biosynthesis genes were shown to be inhibited with staurosporin and MAPK inhibitors. Transcript regulation studies and kinase assays indicated involvement of OsMKK6 in the process. Transgenic rice overexpressing constitutive active *OsMKK6*^*EE*^ exhibited higher expression of genes of PA biosynthesis pathway upon UV stress and also upon infection with *M. oryzae*.

**Conclusion:**

These results suggest a key role of OsMKK6 in regulation of UV responsive expression of genes of PA biosynthesis in rice. This study will help to elucidate the intricate signalling components of UV leading to phytoalexins biosynthesis in rice.

**Electronic supplementary material:**

The online version of this article (doi:10.1186/1939-8433-6-35) contains supplementary material, which is available to authorized users.

## Background

In rice UV radiation leads to accumulation of phytoalexins (PA) which are typically accumulated when rice (*Oryza sativa*) plants are infected with rice blast pathogen *Magnaporthe oryzae* (Cartwright et al. 1981; Kodama et al. 1988). Most of the rice phytoalexins are diterpenoid in nature and fourteen of such compounds have been identified in rice leaves/cells in response to infection by blast pathogen *Magnaporthe oryzae*, elicitors and UV irradiations. These phytoalexins have been grouped into four distinct types of polycyclic diterpene based on the structures of their diterpene hydrocarbon precursors: phytocassanes A–E, oryzalexins A–F, momi-lactones A and B, and oryzalexin S (Additional file 1: Figure S1) (Shimura et al. 2007; Ahuja et al. 2012). Among the rice PA, momilactones are considered to be the major constituents (Cartwright et al. 1981; Kodama et al. 1988,1992). Rice phytoalexins were shown to have anti-fungal activities and their involvement in plant disease resistance have also been proposed (Dillon et al. 1994Peters 2006; Hasegawa et al. 2010). Although PA induction in response to microbe associated molecular pattern (MAMP) has been investigated recently (Kurusu et al. 2010; Kishi-Kaboshi et al. 2010), the signalling mechanism by UV induced phytoalexin accumulation is largely unknown. Here, the role of MAPK signalling cascade in particular of OsMKK6, in UV induced regulation of genes in phytoalexins biosynthesis in rice leaves has been investigated.

## Results and discussion

### UV induces expression of phytoalexin biosynthetic pathway genes and *OsMKK6*

**Figure 1:**
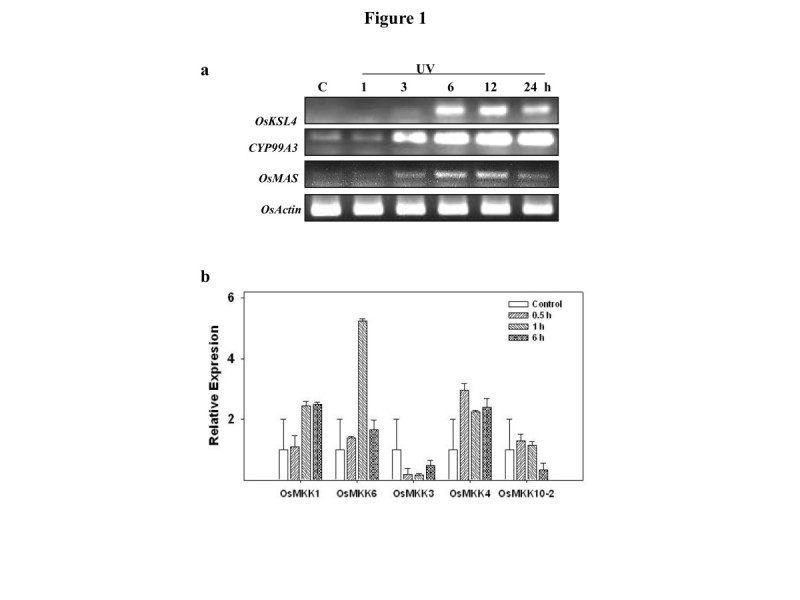
**Expression pattern of genes involved in momilactone biosynthesis and** ***MAPKKs*** **upon UV exposure. a** UV induced expression pattern of genes involved in Phytoalexin biosynthesis, *OsKSL4*, *CYP99A3* and *OsMAS* in rice leaves as shown by RT-PCR analysis. Expression of *Actin* was used as RNA loading control. **b** Analysis of expression levels of *MAPKKs* in rice leaves upon UV exposure by qRT-PCR. Expression levels were normalized against rice actin gene as an internal control and are shown relative to UV unexposed control. The relative level of each gene in control plants at time 0 was standardized as 1. Values are presented as the mean and the error bars indicate SD of three independent experiments.

The expression of PA genes namely, syn-pimara 7,15-diene synthase/kaurene synthase 4 (*OsKSL4*), cytochrome P450 monooxygenase (*CYP99A3*) and momilactone-A synthase (*OsMAS*) were induced upon UV elicitation in Pusa Basmati 1 (Figure 1a) with maximum expression at 6 and 12 hours post treatment.

Mitogen-activated protein kinase (MAPK) signalling cascade is evolutionarily conserved among eukaryotes and is known to have important functions in regulating stress responses (Suarez-Rodriguez et al. 2010; Rao et al. 2011; Sinha et al. 2011; Raina et al. 2012). Mitogen-activated protein kinase kinase (MAPKK), a component of MAPK cascade is believed to be a point of signal convergence and thus acts as a key component of MAPK cascade regulating various stress responses (Suarez-Rodriguez et al. 2010; Kumar et al. 2012). Since regulation of MAPK components also occur at transcriptional level (Morris 2001Kumar et al. 2008), the expression profile of rice *MAPKKs* was studied upon UV elicitation. The maximum UV responsive expression was observed for *OsMKK6* followed by *OsMKK4* and *OsMKK1* (Figure 1b).

### OsMKK6 is phosphorylated in response to UV in rice leaves

**Figure 2:**
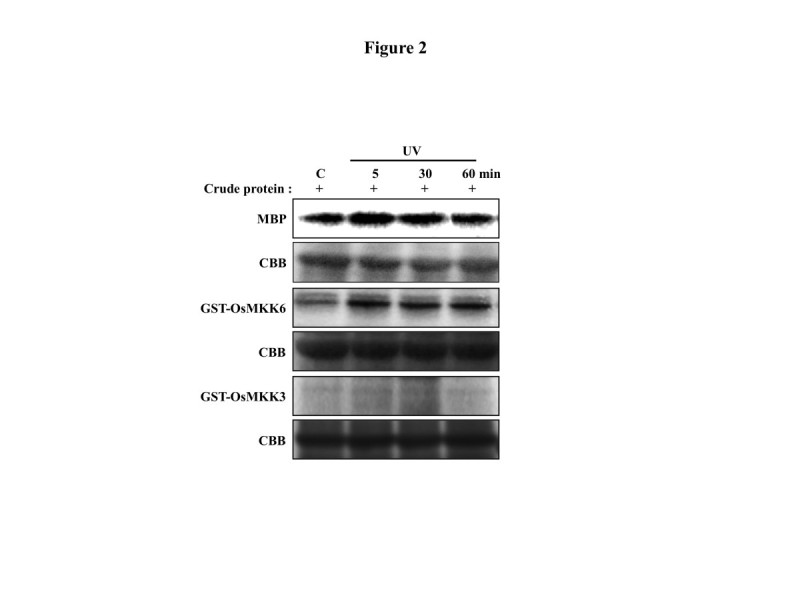
**Phosphorylation of OsMKK6 in response to UV in rice leaves.** Bacterially expressed and purified GST-OsMKK6, GST-OsMKK3 were used as substrates for plant protein extract along with MBP in in-solution kinase assay in the presence of kinase reaction buffer and radiolabelled ATP. CBB stained proteins are shown as equal loading control.

An in-solution kinase assay performed using myelin basic protein (MBP) as an artificial substrate showed activation of MAPK in response to UV (Figure 2). Since, *OsMKK6* showed UV induced expression pattern, UV induced upstream kinase activity for OsMKK6 was checked. GST-OsMKK6 fusion protein was used as a substrate in an in-solution kinase assay with crude protein extract from UV irradiated rice plants. Increased phosphorylation of GST-OsMKK6 upon UV elicitation (Figure 2) indicates its involvement in UV stress. As a control GST-OsMKK3 showed no phosphorylation under similar conditions (Figure 2).

### Specific kinase inhibitors suppress UV induced expression of PA genes in rice leaves

To establish a relationship between MAPK and up-regulated PA biosynthesis genes pharmacological experiment was employed using staurosporin and MAPK cascade specific inhibitors (U0126, PD169316 and SB202190). U0126 blocks MAPKK activation whereas PD169316 and SB202190 block MAPK activation (Suarez-Rodriguez et al. 2010).

**Figure 3:**
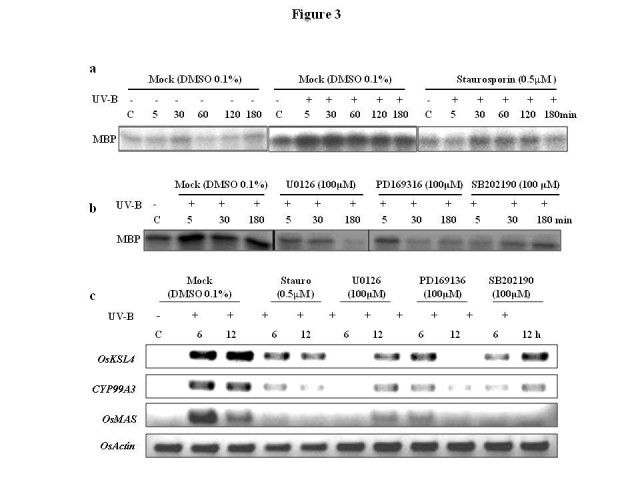
**Staurosporin and MAPK cascade specific inhibitors attenuate UV induced kinase activity and PA gene expression.** Rice plants were pre-treated with inhibitors and irradiated with UV. As a control one set of plants were pre-treated with 0.1% DMSO and irradiated with UV or left unirradiated. **a** Effect of staurosporin on UV induced MAPK activity. **b** Effect of U0126, PD169316 and SB202190 on UV induced MAPK activity. Kinase activity was assayed by performing in-solution kinase assay using MBP as substrate. **c** Effect of staurosporin, U0126, PD169316 and SB202190 on UV induced transcripts accumulation of genes of PA biosynthesis. Transcripts accumulation of *OsKSL4*, *CYP99A3* and *OsMAS* was studied by RT-PCR. Expression of rice actin gene used as a loading control.

As shown in Figure 3a, higher MBP phosphorylation in rice leaves in response to UV irradiation was clearly inhibited by staurosporin. Similarly MAPK cascade specific inhibitors, U0126, PD169316 and SB202190 showed inhibition of UV induced MBP phosphorylation activity in rice leaves (Figure 3b).

Further, this approach was also used to assess the involvement of MAPK cascade in UV induced expression of genes in PA biosynthesis. The UV induced expression of *OsKSL4*, *CYP99A3* and *OsMAS* was found to be reduced in inhibitors fed plants (Figure 3c) indicating involvement of MAPK cascade in UV induced PA accumulation. There were slight differences in inhibition of MBP phosphorylation activity in different time points (such as in U0126, SB202190), showing near complete inhibition in some case to relatively less in the other. The variations observed in MBP phosphorylation activity at different time points could be attributed to the use of ‘seedlings’ for inhibitor treatments as against ‘cell cultures’ which appears to respond more uniformly to such inhibitor treatments (Ramani and Chelliah, 2007). Further, differential uptake of inhibitors by plants might also be partly responsible for differences in inhibition pattern.

### Transgenic rice overexpressing *OsMKK6*^*EE*^exhibits more pronounced effect on UV and blast inducible expression of PA biosynthesis genes

In order to investigate the direct involvement of OsMKK6 in UV inducible expression of genes in PA biosynthesis, transgenic rice lines overexpressing constitutively active form of OsMKK6 (OsMKK6^EE^) were generated. The expression of *OsMKK6*^*EE*^ was driven in transgenic lines by CaMV 35S promoter (Additional file 2: Figure S2a-f). Two homozygous *OsMKK6*^*EE*^*-* overexpression lines (*OsMKK6*^*EE*^-10 and *OsMKK6*^*EE*^-18) in their T3 generation were used for the study. Expression levels of *OsMKK6* were checked in three weeks old transgenic plants by qRT-PCR. *OsMKK6*^*EE*^-10 and *OsMKK6*^*EE*^-18 lines showed ~7 fold and ~4 fold increased *OsMKK6* transcript levels, respectively as compared to wild type plants (Additional file 2: Figure S2g). These lines were used to investigate the effect of *OsMKK6*^*EE*^ over expression, on UV inducible expression pattern of genes involved in PA biosynthesis.

**Figure 4:**
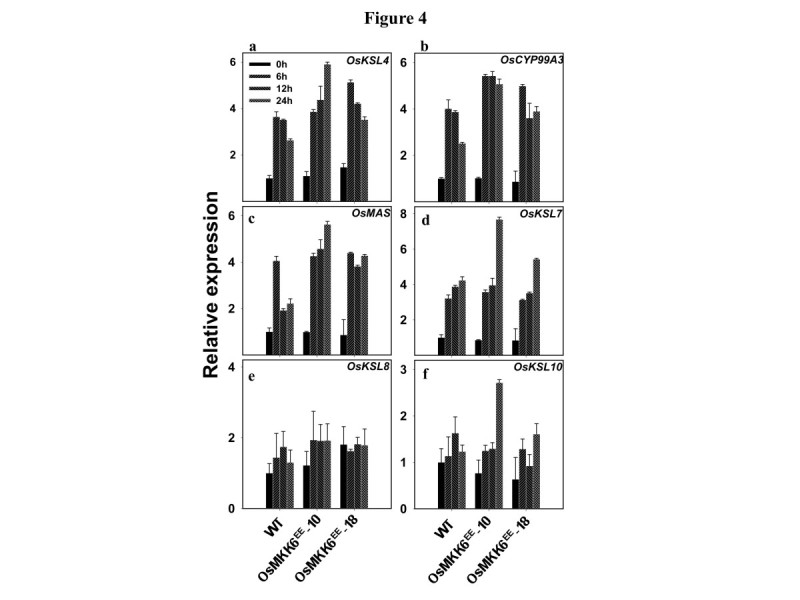
**Involvement of OsMKK6 in UV induced expression of genes of phytoalexin biosynthesis in rice leaves.** Expressions of **a** *OsKSL4*, **b** *OsCYP99A3*, **c** *OsMAS*, **d** *OsKSL7*, **e** *OsKSL8*, and **f** *OsKSL10* in *OsMKK6*^*EE*^ overexpression lines was checked by qRT-PCR. Expression levels were normalized against rice actin gene as an internal control and are shown relative to UV-B unexposed control. The relative level of each gene in control plants at time 0 was standardized as 1. Values are presented as the mean and the error bars indicate SD of three independent experiments.

Expression patterns of the six genes (*OsKSL4*, *CYP99A3*, *OsMAS*, *OsKSL7*, *OsKSL8* and *OsKSL10*) (Additional file 1: Figure S1) involved in PA biosynthesis were checked in transgenic plants upon UV elicitation. The qRT-PCR analyses revealed higher expression of *OsKSL4*, *CYP99A3*, *OsMAS* and *OsKSL7* genes in *OsMKK6*^*EE*^-10 and *OsMKK6*^*EE*^-18 plants as compared to wild type plants (Figure 4a-d). *OsKSL8* and *OsKSL10* showed only a slight increase in expression than the control plants in transgenic plants in response to UV (Figure 4e-f). The results indicate that under UV stress OsMKK6 may have role in regulation of biosynthesis of momilactone A-B, phytocassanes A-E and have little or no involvement in accumulation of oryzalexin A-F and oryzalexin S.

**Figure 5:**
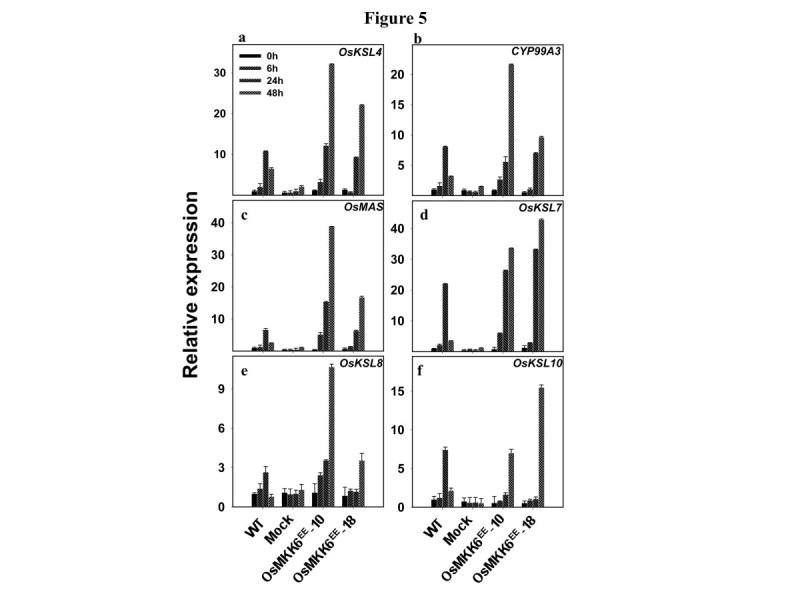
**Involvement of OsMKK6 in rice blast (** ***M. oryzae)*** **induced expression of genes of phytoalexin biosynthesis.** Expressions of **a** *OsKSL4*, **b** *OsCYP99A3*, **c** *OsMAS*, **d** *OsKSL7*, **e** *OsKSL8*, and **f** *OsKSL10* in *OsMKK6*^*EE*^ overexpression lines were checked by qRT-PCR. Plants were inoculated with spore suspension of *M. oryzae* or mock inoculated to wild type plants. Expression levels were normalized against rice actin gene as an internal control and are shown relative to uninoculated control WT plant. The relative level of each gene in control plants at time 0 was standardized as 1. Values are presented as the mean and the error bars indicate SD of three independent experiments. Legends for the bars showns on top, left panel is same for all graphs.

Phytoalexins are also accumulated in rice leaves after infection by the blast fungus, *Magnaporthe oryzae* (Cartwright et al. 1981; Peters 2006). Further, expression analysis of all rice *MAPKKs* in Massively Parallel Signature Sequencing (MPSS) (Brenner et al. 2000) database revealed the increase in expression levels of *OsMKK6* and *OsMKK10-2* in different libraries post *M. oryzae* infection (Additional file 3: Figure S3). *OsMKK6*^*EE*^ over-expressing lines were therefore used to investigate a possible role of OsMKK6 in regulating expression of PA genes upon infection by blast fungus. qRT-PCR analyses revealed, enhanced expression of all the six PA genes in *OsMKK6*^*EE*^-10 and *OsMKK6*^*EE*^-18 (Figure 5a-f). These results suggest a positive effect of *OsMKK6*^*EE*^ overexpression on UV and blast induced transcripts accumulation of genes in PA biosynthesis. Unlike UV, higher expression of all six PA genes after blast infection in *OsMKK6*^*EE*^ overexpressing plants suggest possible role of OsMKK6 in accumulation of all diterpenoid phytoalexins upon blast infection. However, it is important to note that higher expressions of genes of PA biosynthesis were observed in *OsMKK6*^*EE*^ overexpressing plants only after UV treatment or blast infection and the over-expression *per se* was not sufficient to enhance expressions of the mentioned genes. This indicates involvement of additional OsMKK6-independent and UV/blast inducible component/s in UV/blast signal transduction and/or regulation of genes of phytoalexin biosynthesis.

In recent years two independent studies have shed light on PA biosynthesis in rice using suspension cultured rice cells and elicitors. Kishi-Kaboshi et al. (2010) have shown role of OsMKK4 in expression of PA genes and biosynthesis of diterpenoid phytoalexins, momilactones and phytocassanes in response to fungal elicitor (N-acetylchitooctaose). Another study (Kurusu et al. 2010) has demonstrated the role of OsCIPK14/15 in regulation of genes in PA biosynthesis and other defense responses induced by fungal elicitor (*Trichoderma viride/ethylene-inducing xylanase* [TvX/EIX]). These two reports and our findings indicate involvement of distinct signalling pathways in regulation of PA biosynthesis in rice depending upon upstream signal. It has been shown that even distinct MAMPs elicit distinct Ca^2+^ signatures in amplitude and duration giving specific responses (Tena et al. 2011). UV and MAMPs are different in nature, therefore, are likely to activate distinct signalling. It is also plausible that the pathways act synergistically in the regulation of PA biosynthesis. Further, it is important to note that both the studies (Kishi-Kaboshi et al. 2010; Kurusu et al. 2010) showing role of OsMKK4-OsMPK3/OsMPK6 and OsCIPK14/15 have been performed in suspension cell cultures which lack cellular differentiation and provide entirely different environmental conditions than naturally grown plants.

Since, there was no inclusion of OsMKK6 in the study by Kishi-Kaboshi et al. (2010), it is not possible to assess contribution of OsMKK6 towards regulation of PA in response to MAMP in rice cells. Similarly, in the present work, study on OsMKK4 was limited only to expression analysis and *OsMKK4* albeit showed higher expression upon UV elicitation.

## Conclusion

To conclude, this study has shown the role of MAPK cascade in particular of the OsMKK6 in the regulation of genes in phytoalexin biosynthesis in response to UV and blast infection.

## Methods

### Plant material, stress and inhibitors treatments

*Oryza sativa* L. indica cultivar group var Pusa Basmati 1 was used in the present study. Plants were grown in growth chamber at 28°C with 16/8 day light condition or in green house at 28°C and three-four week old plants were used for the experiments. Transgenic plants were germinated and grown on hygromycin media for two weeks then shifted to green house. UV treatment was given by exposing three-four week old rice seedlings to UV-B tubes (Phillips, Netherland) for 10 minutes. Inoculation of *Magnaporthe oryzae- M. oryzae* virulent strain (*M. oryzae* Dehradun isolate) was procured from National Research Centre for Plant Biotechnology, New Delhi. Fungal spore inoculation was followed as previously described (Reyna and Yang 2006). For inhibitor treatments, individual plants were incubated with inhibitors (Staurosporin, 0.5 μM; U0126, 100 μM; PD169316, 100 μM; and SB202190, 100 μM) in 2.0 ml tubes for four hours before UV treatment. The inhibitor fed plants were then UV irradiated. For mock treatment, plants were treated with solvent (DMSO 0.1) at final concentration of 0.1%.

The generation of rice transgenics overexpressing OsMKK6^EE^ is being described in Additional file 2: Figure S2.

### RT–PCR analysis, Kinase assay

The qRT-PCR was carried out in 48/96/384 well plate ABI Prism 7000 sequence detection system (Applied Biosystems, City, CA) as mentioned in previous report (Jaggi et al., 2011). Protein extraction and kinase assay were performed as mentioned previously (Rao et al., 2011). Semi-quantitative RT-PCR (sqRT-PCR) was performed following standard PCR conditions and optimal cycle number 26-30 in 50μl reaction volume. PCR amplification of Actin was used as control to ensure an equal cDNA. Amplification was carried out in iCyclerTM (BIO-RAD). The PCR product (50 μl) was loaded onto 1.5% agarose/EtBr gel and visualised.

## Electronic supplementary material

Additional file 1: Figure S1: Phytoalexin biosynthetic pathway in rice. Enzymes whose gene expressions are studied in the present study are shown in bold font. Arrows facing upward indicate steps regulated by OsTGAP1 (Okada et al. 2009). Dashed arrows show involvement of multiple steps. (PDF 101 KB)

Additional file 2: Figure S2: Generation and analyses of *OsMKK6*^*EE*^ transgenic rice. **a** PCR amplification of full length *OsMKK6* mutated clone (*OsMKK6*^*EE*^) using specific primers having adapter sequences to clone in pCAMBIA1303. **b** Colony PCR of 5 randomly selected transformed bacterial colonies with gene specific primer pairs from the ends. **c** Restriction digestion of plasmid DNA isolated from positive colony with *Nco* I and *Bgl* II. **d** T-DNA in binary vector pCAMBIA1303 containing full length *OsMKK6*^*EE*^ at *Nco* I/*Bgl* II sites. **e** Screening of putative transgenic lines for the presence of *OsMKK6* transgene by total genomic PCR using pCAMBIA specific forward and OsMKK6 specific reverse primer. ‘C’ and M denotes control plant (wild type) and DNA ladder (500 bp) respectively. **f** Northern blot analysis showing expression of *OsMKK6* in control and transgenic plants. **g** qRT-PCR analysis to study expression of *OsMKK6* in *OsMKK6*^*EE*^ overexpression lines T3 generation and control plants. Expression levels were normalized against rice actin gene as an internal control and are shown relative to wild type. The relative level of *OsMKK6* expression in wild type plants was standardized as 1. Values are presented as the mean and the errors bars indicate standard deviation of three independent experiments. (PDF 75 KB)

Additional file 3: Figure S3: Transcript abundance of rice MAPKKs in *Magnaporthe* treated Nipponbare shoots libraries from MPSS database. Transcripts abundance was shown post 3 h, 6 h, 12 h, 24 h, 48 h, 96 h of *Magnaporthe* treatment (MS-3 to MS-96). MC00 and MC24 represent mock treated samples at 0 h and 24 h of treatment respectively. (PDF 39 KB)

Below are the links to the authors’ original submitted files for images.

Authors’ original file for figure 1

Authors’ original file for figure 2

Authors’ original file for figure 3

Authors’ original file for figure 4

Authors’ original file for figure 5

Authors’ original file for figure 6

Authors’ original file for figure 7

## References

[CR1] Ahuja I, Kissen R, Bones AM (2012). Phytoalexins in defense against pathogens. Trends Plant Sci.

[CR2] Brenner S, Johnson M, Bridgham J (2000). Gene expression analysis by massively parallel signature sequencing (MPSS) on microbead arrays. Nat Biotechnol.

[CR3] Cartwright DW, Langcake P, Pryce RJ, Leworthy DP, Ride JP (1981). Isolation and characterization of two phytoalexins from rice as momilactones A and B. Phytochemistry.

[CR4] Dillon VM, Overton J, Grayer RJ, Harborne JB (1994). Differences in phytoalexin response among rice cultivars of different resistance to blast. Phytochemistry.

[CR5] Hasegawa M, Mitsuhara I, Seo S, Imai T, Koga J, Okada K, Yamane H, Ohashi Y (2010). Phytoalexin accumulation in the interaction between rice and the blast fungus. Mol Plant Microbe Interact.

[CR6] Jaggi M, Kumar S, Sinha AK (2011). Overexpression of an apoplastic peroxidase gene CrPrx in transgenic hairy root lines of Catharanthus roseus. Appl Microbiol Biotech.

[CR7] Kishi-Kaboshi M, Okada K, Kurimoto L (2010). A rice fungal MAMP responsive MAPK cascade regulates metabolic flow to antimicrobial metabolite synthesis. Plant J.

[CR8] Kodama O, Miyakawa J, Akatsuka T, Kiyosawa S (1992). Sakuranetin, a flavanone phytoalexin from ultraviolet-irradiated rice leaves. Phytochemistry.

[CR9] Kodama O, Suzuki T, Miyakawa J, Akatsuka T (1988). Ultraviolet-induced accumulation of phytoalexins in rice leaves. Agric Biol Chem.

[CR10] Kumar K, Rao KP, Sharma P, Sinha AK (2008). Differential regulation of rice mitogen activated protein kinase kinase (MKK) by abiotic stress. Plant Physiol Biochem.

[CR11] Kumar K, Wankhede DP, Sinha AK (2012). Signal convergence through the lenses of MAP kinases: paradigms of stress and hormone signaling in plants Front. Biol.

[CR12] Kurusu T, Hamada J, Nokajima H (2010). Regulation of microbe-associated molecular pattern-induced hypersensitive cell death, phytoalexin production, and defense gene expression by calcineurin B-like protein-interacting protein kinases, OsCIPK14/15, in rice cultured cells. Plant Physiol.

[CR13] Morris PC (2001). MAP kinase signal transduction pathways in plants. New Phytologist.

[CR14] Okada A, Okada K, Miyamoto K, Koga J, Shibuya N, Nojiri H, Yamane H (2009). OsTGAP1, a bZIP transcription factor, coordinately regulates the inductive production of diterpenoid phytoalexins in rice. J Biol Chem.

[CR15] Peters RJ (2006). Uncovering the complex metabolic network underlying diterpenoid phytoalexin biosynthesis in rice and other cereal crop plants. Phytochemistry.

[CR16] Ramani S, Chelliah J (2007). UV-B-induced signaling events leading to enhanced production of catharanthine in *Catharanthus roseus* cell suspension cultures. BMC Plant Biol.

[CR17] Raina SK, Wankhede DP, Jaggi M, Singh P, Jalmi SK, Raghuram B, Sheikh AH, Sinha AK (2012). CrMPK3, a mitogen activated protein kinase from *Catharanthus roseus* and its possible role in stress induced biosynthesis of monoterpenoid indole alkaloids. BMC Plant Biol.

[CR18] Rao KP, Vani G, Kumar K, Wankhede DP, Mishra M, Gupta M, Sinha AK (2011). Arsenic stress activates MAP kinase in rice roots and leaves. Arch Biochem Biophys.

[CR19] Reyna NR, Yang Y (2006). Molecular analysis of the rice MAP Kinase gene family in relation to *Magnaporthe grisea* infection. Mol Plant Microbe Interact.

[CR20] Shimura K, Okada A, Okada K, Jikumaru Y, Ko KW, Toyomasu T, Sassa T, Hasegawa M, Kodama O, Shibuya N, Koga J, Nojiri H, Yamane H (2007). Identification of a biosynthetic gene cluster in rice for momilactones. J Biol Chem.

[CR21] Sinha AK, Jaggi M, Raghuram B, Tuteja N (2011). Mitogen-activated protein kinase signalling in plants under abiotic stress. Plant Signal Behav.

[CR22] Suarez-Rodriguez MC, Petersen M, Mundy J (2010). Mitogen-Activated Protein Kinase signalling in plants. Annu Rev Plant Biol.

[CR23] Tena G, Boudsocq M, Sheen J (2011). Protein kinase signaling networks in plant innate immunity. Curr Opin Plant Biol.

