# Supplementary material for: Involvement of mitogen activated protein kinase kinase 6 in UV induced transcripts accumulation of genes in phytoalexin biosynthesis in rice
Source: Rice (N Y). 2013 Dec 2;6:35. doi: 10.1186/1939-8433-6-35 (PMC4883730; doi:10.1186/1939-8433-6-35)
Supplement: Supplementary file 1 — Additional file 1: Figure S1: Phytoalexin biosynthetic pathway in rice. Enzymes whose gene expressions are studied in the present study are shown in bold font. Arrows facing upward indicate steps regulated by OsTGAP1 (Okada et al. 2009). Dashed arrows show involvement of multiple steps. (PDF 101 KB) [file 12284_2012_67_MOESM1_ESM.pdf]

Additional file 1

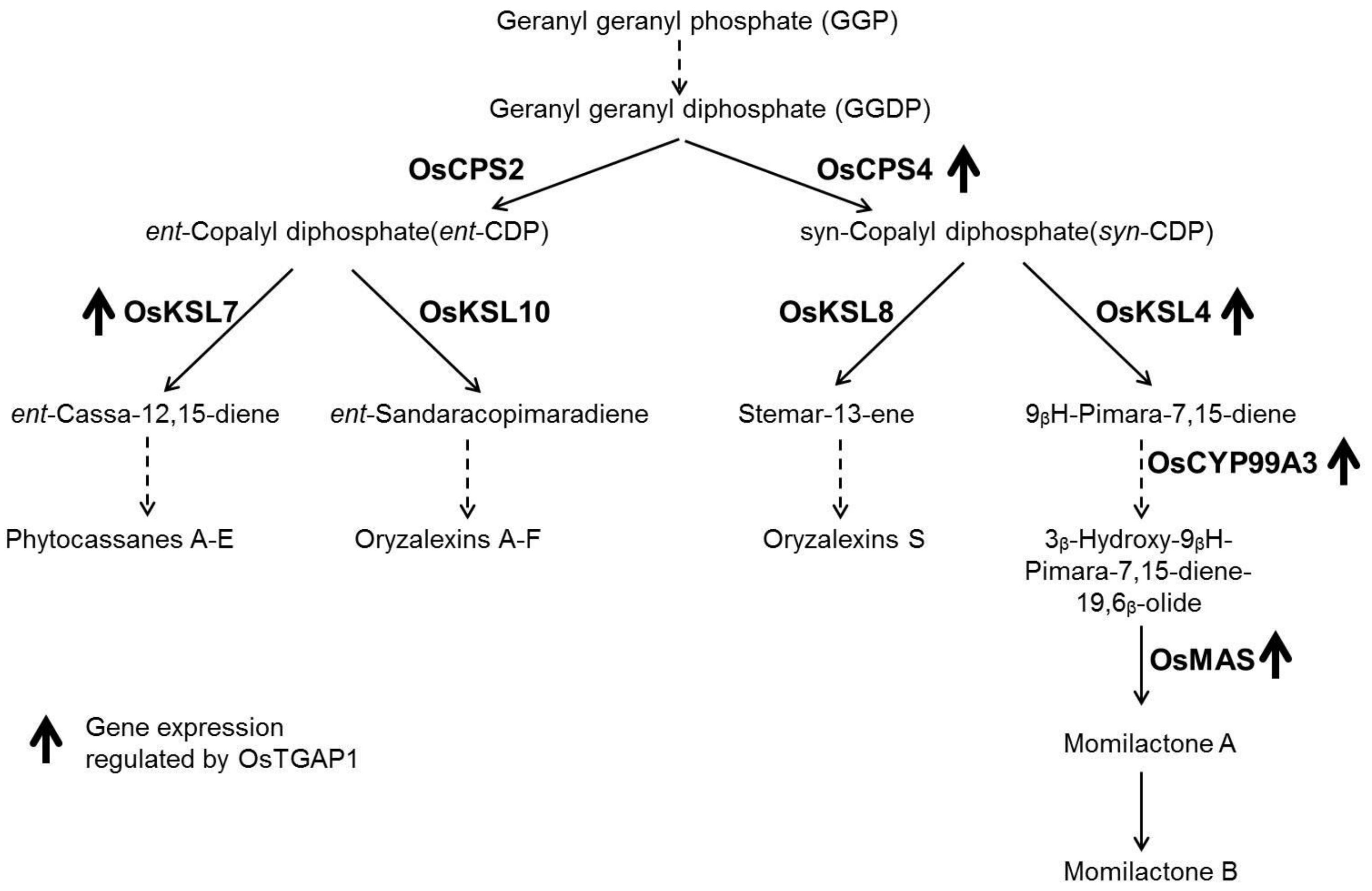

Supplemental Figure S1

Reference:
Okada A, Okada K, Miyamoto K, Koga J, Shibuya N, Nojiri H, and Yamane H (2009) OsTGAP1, a bZIP transcription factor, coordinately regulates the inductive production of diterpenoid phytoalexins in rice. J Biol Chem 284:26510-18
