# Supplementary figures and images for: Involvement of mitogen activated protein kinase kinase 6 in UV induced transcripts accumulation of genes in phytoalexin biosynthesis in rice
Source: Rice (N Y). 2013 Dec 2;6:35. doi: 10.1186/1939-8433-6-35 (PMC4883730; doi:10.1186/1939-8433-6-35)

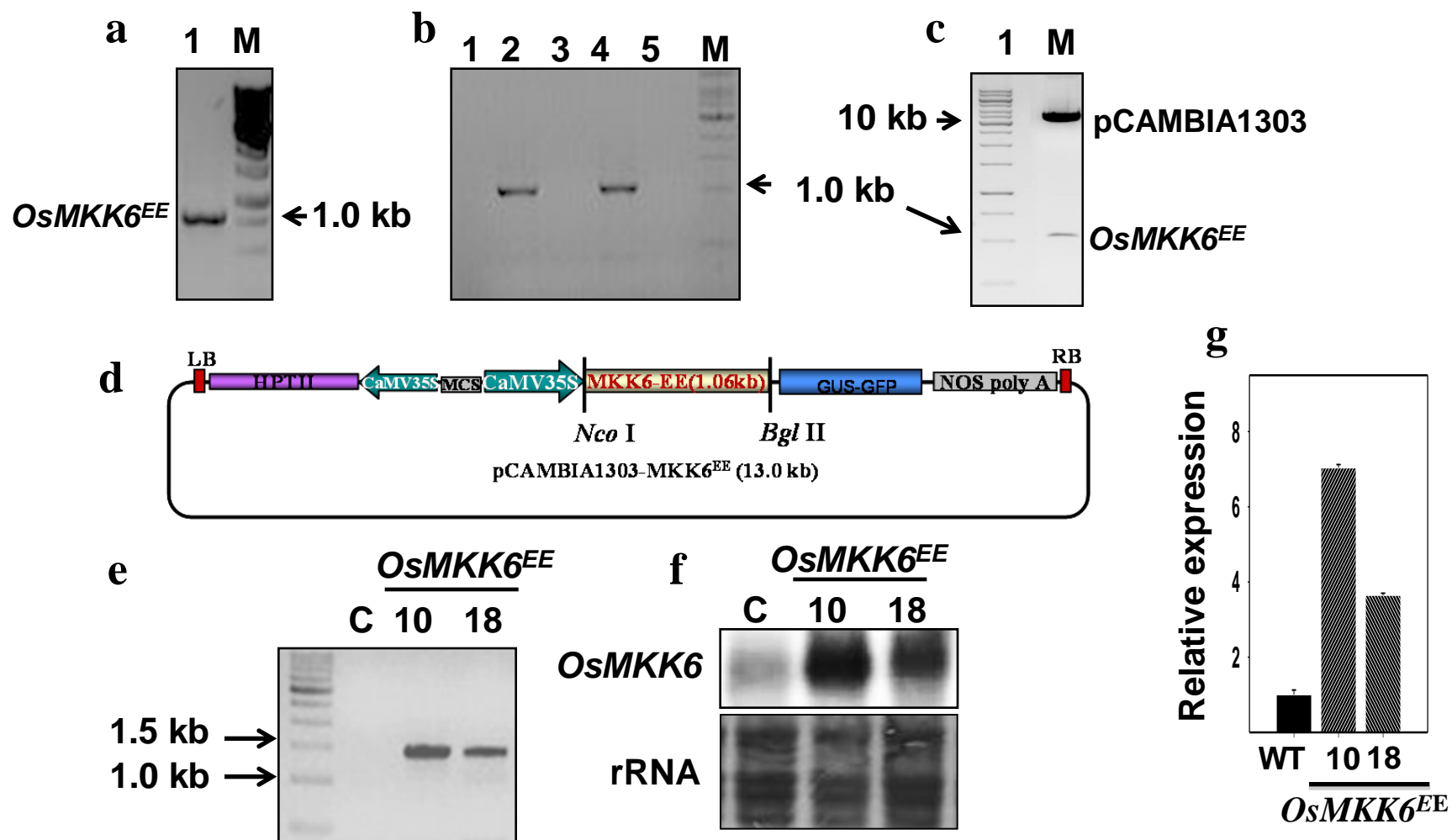

Supplemental Figure S2

Supplement: Supplementary file 2 — Additional file 2: Figure S2: Generation and analyses of OsMKK6EE transgenic rice. a PCR amplification of full length OsMKK6 mutated clone (OsMKK6EE) using specific primers having adapter sequences to clone in pCAMBIA1303. b Colony PCR of 5 randomly selected transformed bacterial colonies with gene specific primer pairs from the ends. c Restriction digestion of plasmid DNA isolated from positive colony with Nco I and Bgl II. d T-DNA in binary vector pCAMBIA1303 containing full length OsMKK6EE at Nco I/Bgl II sites. e Screening of putative transgenic lines for the presence of OsMKK6 transgene by total genomic PCR using pCAMBIA specific forward and OsMKK6 specific reverse primer. ‘C’ and M denotes control plant (wild type) and DNA ladder (500 bp) respectively. f Northern blot analysis showing expression of OsMKK6 in control and transgenic plants. g qRT-PCR analysis to study expression of OsMKK6 in OsMKK6EE overexpression lines T3 generation and control plants. Expression levels were normalized against rice actin gene as an internal control and are shown relative to wild type. The relative level of OsMKK6 expression in wild type plants was standardized as 1. Values are presented as the mean and the errors bars indicate standard deviation of three independent experiments. (PDF 75 KB) [file 12284_2012_67_MOESM2_ESM.pdf]

## Additional file 3

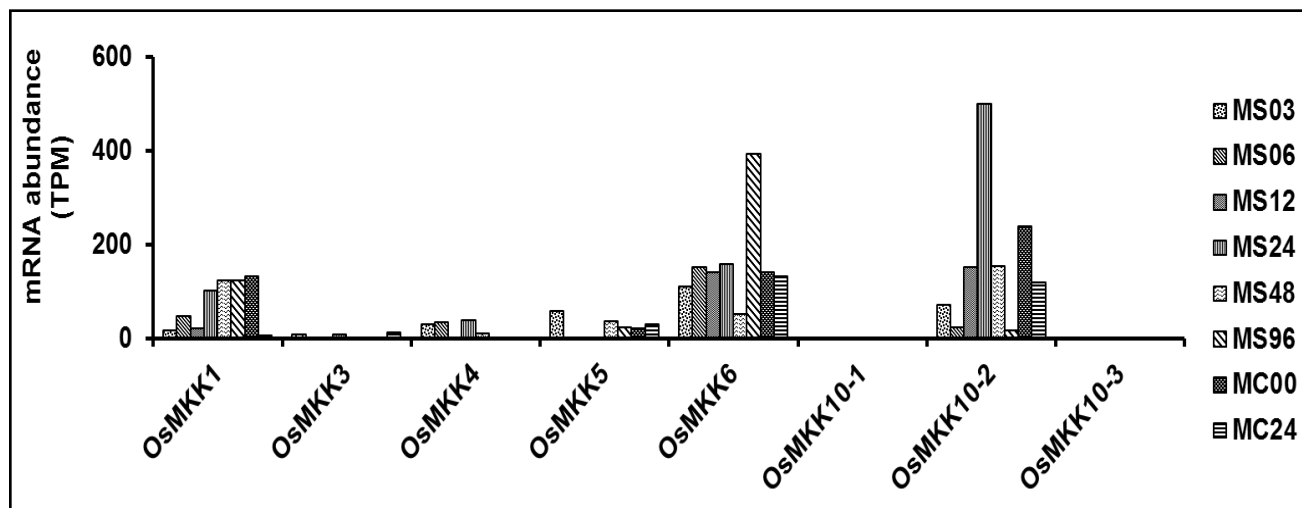

Supplemental Figure S3

Supplement: Supplementary file 3 — Additional file 3: Figure S3: Transcript abundance of rice MAPKKs in Magnaporthe treated Nipponbare shoots libraries from MPSS database. Transcripts abundance was shown post 3 h, 6 h, 12 h, 24 h, 48 h, 96 h of Magnaporthe treatment (MS-3 to MS-96). MC00 and MC24 represent mock treated samples at 0 h and 24 h of treatment respectively. (PDF 39 KB) [file 12284_2012_67_MOESM3_ESM.pdf]

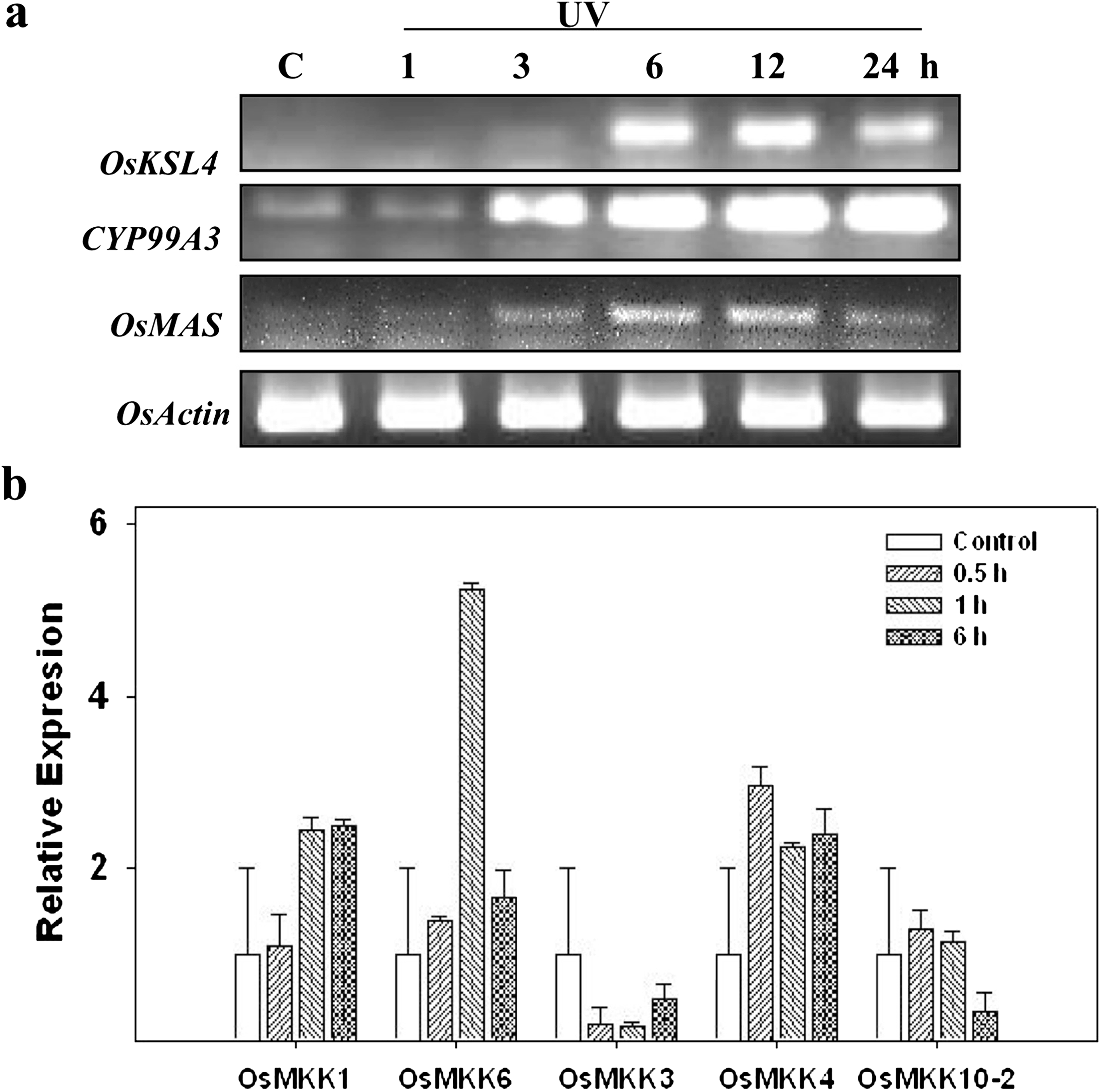

Supplement: Supplementary file 4 — Authors’ original file for figure 1 [file 12284_2012_67_MOESM4_ESM.tif]

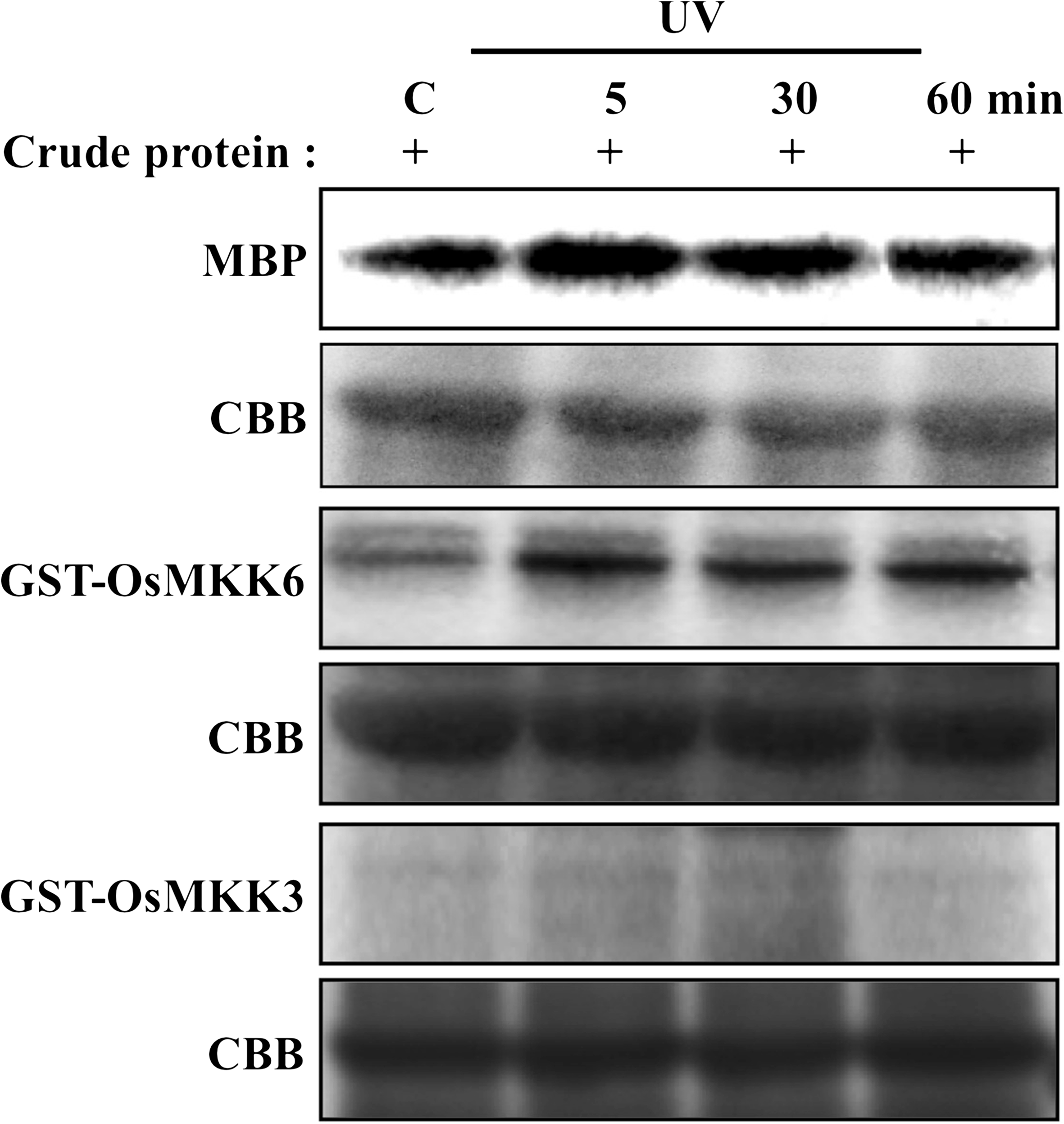

Supplement: Supplementary file 5 — Authors’ original file for figure 2 [file 12284_2012_67_MOESM5_ESM.tif]

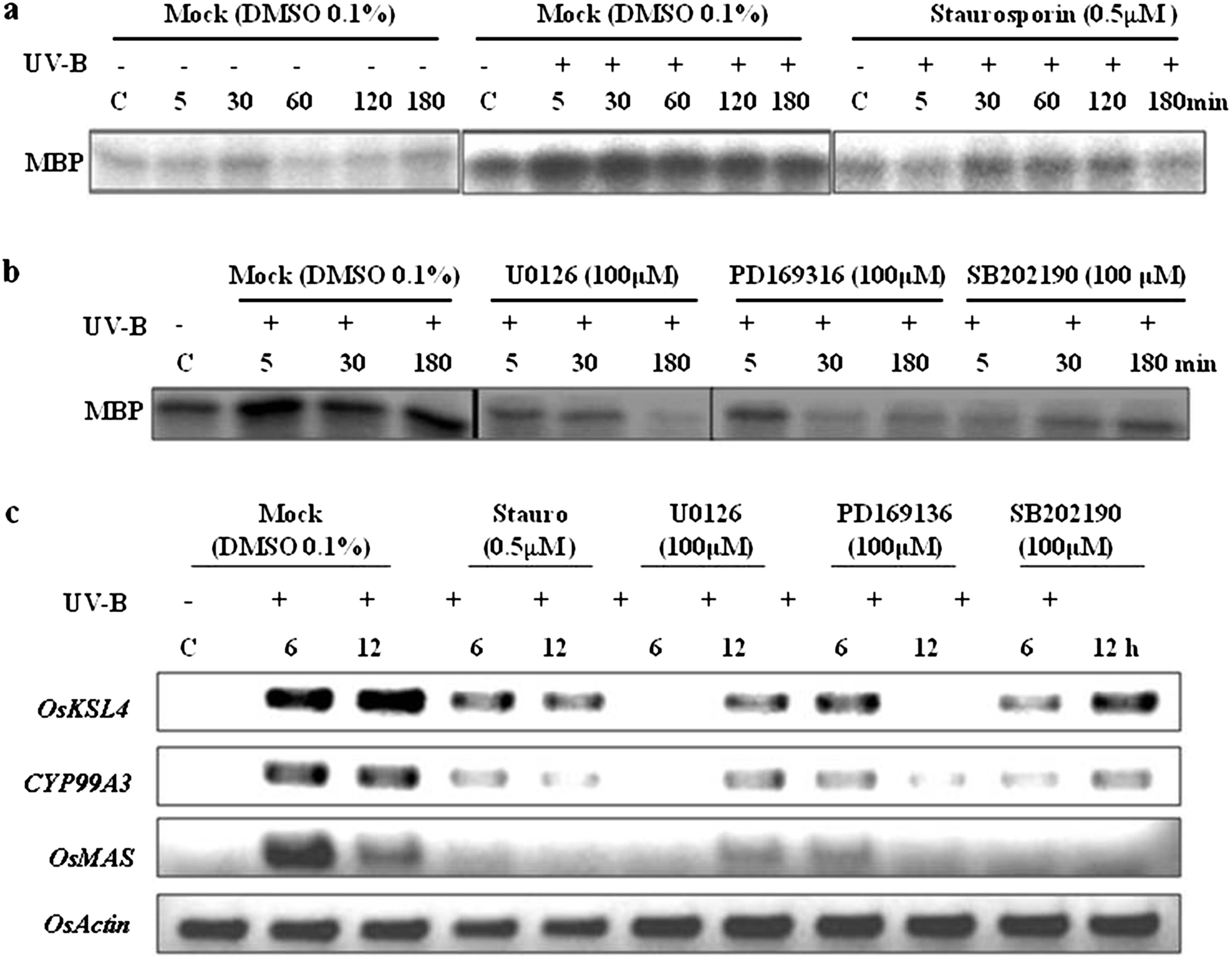

Supplement: Supplementary file 6 — Authors’ original file for figure 3 [file 12284_2012_67_MOESM6_ESM.tif]

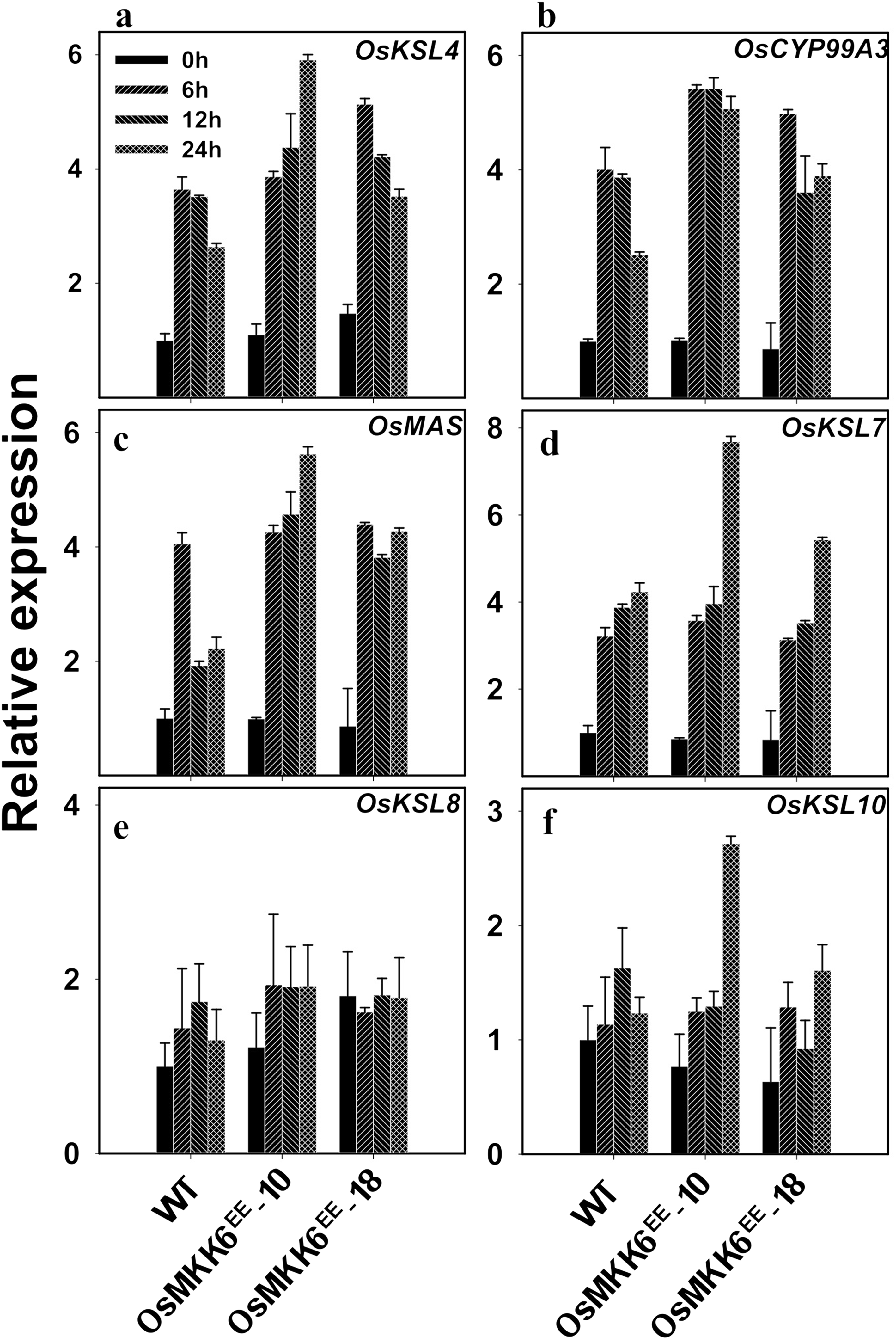

Supplement: Supplementary file 7 — Authors’ original file for figure 4 [file 12284_2012_67_MOESM7_ESM.tif]

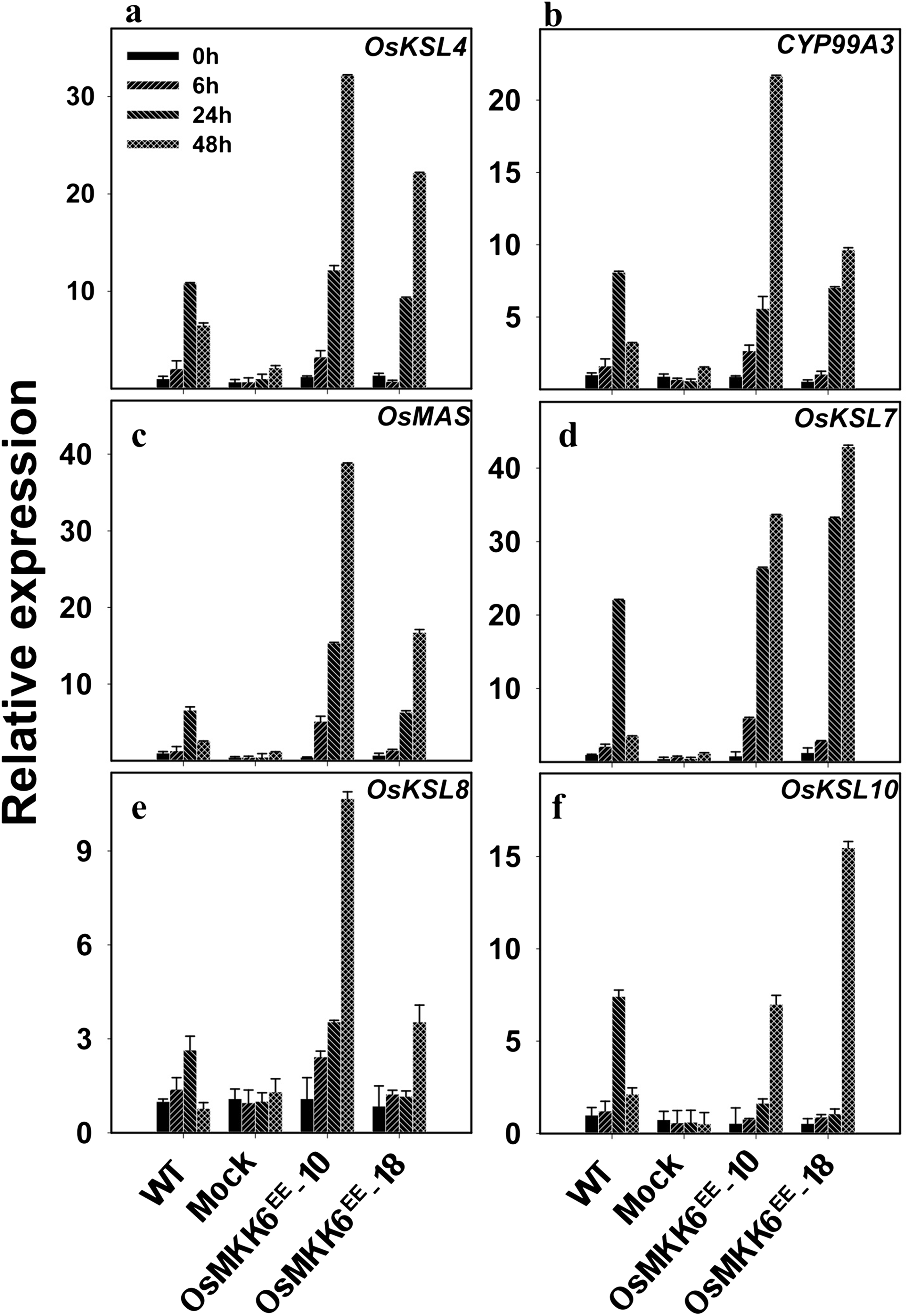

Supplement: Supplementary file 8 — Authors’ original file for figure 5 [file 12284_2012_67_MOESM8_ESM.tif]

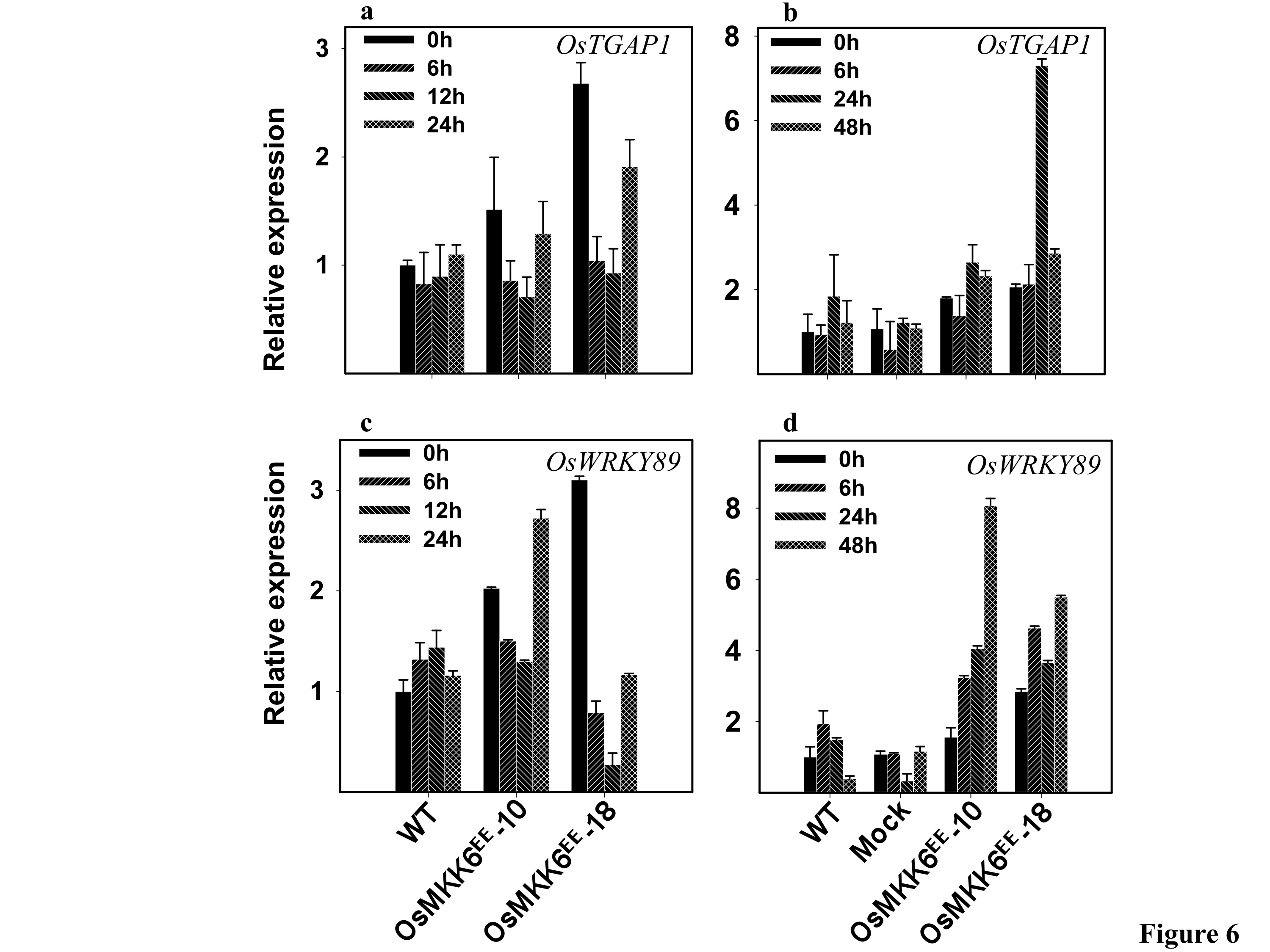

Supplement: Supplementary file 9 — Authors’ original file for figure 6 [file 12284_2012_67_MOESM9_ESM.jpeg]

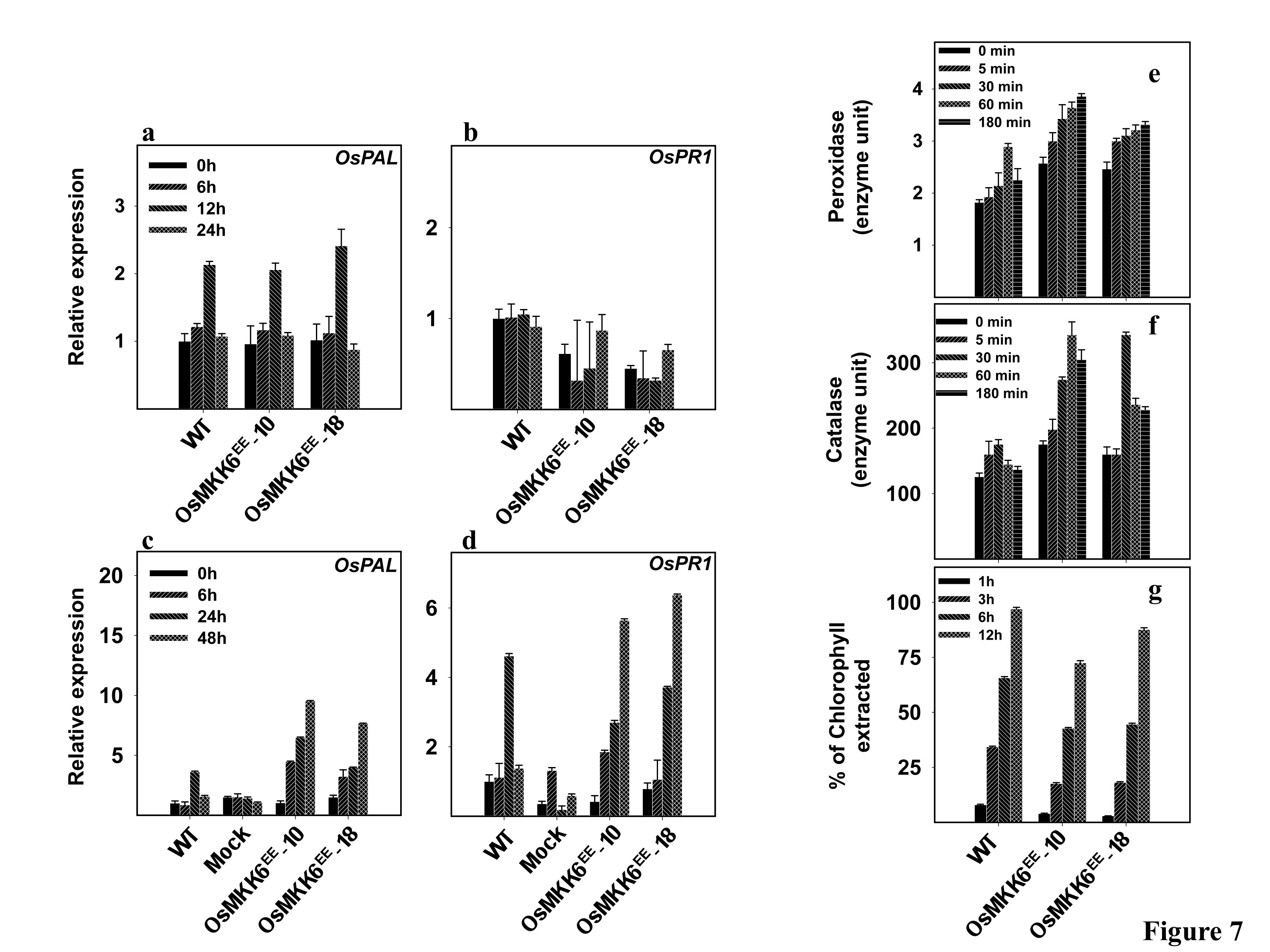

Supplement: Supplementary file 10 — Authors’ original file for figure 7 [file 12284_2012_67_MOESM10_ESM.jpeg]
